# Serum NF-κB in Epstein–Barr Virus-Related Oropharyngeal Carcinoma Diagnostic Usability

**DOI:** 10.3390/cancers16132328

**Published:** 2024-06-25

**Authors:** Anna Polz, Kamal Morshed, Bartłomiej Drop, Małgorzata Polz-Dacewicz

**Affiliations:** 1 Genomed S.A., 02-971 Warsaw, Poland; anna.polz@genomed.pl; 2 Department of Otolaryngology Head and Neck Cancer, Casemiro Pulaski Radom University, 26-600 Radom, Poland; k.morshed@uthrad.pl; 3 Department of Computer Science and Medical Statistics with the e-Health Laboratory, Medical University of Lublin, 20-090 Lublin, Poland; bartlomiej.drop@umlub.pl; 4 Department of Virology with Viral Diagnostics Laboratory, Medical University of Lublin, 20-093 Lublin, Poland

**Keywords:** NF-κB, oropharyngeal cancer, EBV, biomarker

## Abstract

**Simple Summary:**

Currently, many researchers are focusing their study on the search for new, non-invasive biomarkers with high diagnostic and prognostic usefulness in head and neck cancers (HNCs). Most of them concern nasopharyngeal cancer (NPC). However, our research team focused on the most common form in our region, i.e., cancer located in the oropharynx. This was the premise for undertaking research assessing the diagnostic usefulness of measuring nuclear factor kappa B (NF-κB) in oropharyngeal squamous cell carcinoma (OPSCC) linked to EBV. Therefore, we assessed the frequency and level of NF-κB in the serum of patients with this cancer, taking into account clinicopathological features, i.e., grading (G1–G3) and TN classification. The obtained results indicate a significantly higher NF-κB level in the advanced clinical stage of cancer. In turn, ROC analysis confirmed the diagnostic accuracy of this protein. It has been shown that the determination of NF-κB may be a useful diagnostic and prognostic marker of OPSCC linked to EBV.

**Abstract:**

Early diagnosis and effective therapy are the fundamental challenge for modern oncology. Hence, many researchers focus on the search for new or improved biomarkers. Due to the great importance of nuclear factor kappa B (NF-κB) in physiological and pathological processes, we focused on assessing its usefulness as a biomarker in OPSCC. The purpose of the research presented here was to evaluate the prevalence and the level of NF-κB in the serum of OPSCC patients (ELISA). Serum NF-κB levels were also assessed depending on the degree of histological differentiation of the tumor and TN classification. Additionally, we considered the existence of a correlation between the concentration of NF-κB and EBV antibody titers, viral load and selected MMPs—MMP3 and MMP9. Taken together, the obtained results demonstrated that NF-κB level was significantly higher among patients with EBV-related OPSCC than among those without EBV. In addition, the level of NF-κB was significantly higher in more advanced clinical stages. Moreover, a positive correlation was found between the concentration of NF-κB and the level of selected EBV antibodies, viral load and both tested MMPs. The diagnostic accuracy of NF-κB was confirmed by ROC analysis.

## 1. Introduction

Due to constantly increasing morbidity and mortality rates, head and neck cancer (HNC) constitutes an important public health problem, especially in the clinical aspect. Therefore, both the diagnosis and therapy of these cancers are an important challenge for modern medicine.

Approximately 5000 new cases of HNCs are diagnosed in Poland each year [1]. Tumors originating from the epithelium of the oral cavity, pharynx or larynx are histologically classified as head and neck squamous cell carcinoma (HNSCC). Many different factors play a role in the etiopathogenesis of these cancers, among which environmental and lifestyle agents have been well-known and documented. Nevertheless, infections caused by oncogenic viruses, especially human papillomavirus (HPV) and Epstein–Barr virus (EBV), play a significant role in the process of carcinogenesis [2,3].

EBV was discovered in 1964 by Epstein, Achong and Barr by electron microscopy in cells derived from Burkitt’s lymphoma [4]. Four years later (1968), it was found to be the etiological agent of infectious mononucleosis. In addition, EBV causes other clinical syndromes—chronic active EBV infection and X-linked lymphoproliferative disease [5]. Then, in 1970, EBV DNA was detected in tissues collected from patients with nasopharyngeal cancer (NPC) [6]. Since then, there has been increasing interest in the role of this virus in the development and progression of various malignancies, including HNCs.

EBV (according to virological taxonomy called human herpes virus 4 (HHV-4)), has been classified in the *Orthoherpesviridae* family, subfamily *Gammaherpesvirinae* [7]. Like other herpesviruses, the virus particle (about 120–180 nm in diameter) contains a double-stranded DNA of approximately 172 kb and 85 genes, a nucleocapsid composed of 162 capsomeres, protein tegument and an envelope with glycoprotein spikes [8].

EBV is one of the most common human viruses worldwide, with the percentage of the infected population estimated at approximately 95% [9].

Primary infection, most often asymptomatic, occurs in childhood because it is spread primarily through saliva and other body fluids. Showing tropism for B lymphocytes and epithelial cells, EBV infects them, causing their proliferation.

Based on extensive scientific evidence, in 1997, the International Agency for Research on Cancer classified EBV as a Group I carcinogen [10]. Thus, EBV became the first human virus capable of transforming infected host cells. Persistent EBV infection may lead not only to the development but also to the progression of cancers. Many cancers associated with EBV infection have been described in the literature [9,11,12,13,14,15,16]. It has been estimated that 1.5% of all human cancers and 1.8% of tumor deaths are associated with persistent EBV infection [17].

The research provided by the world’s scientific literature focuses mainly on NPC, which occurs most often in people living in Asia [18]. Unfortunately, few studies have been conducted in other populations with a low incidence of this type of cancer [3]. As Globocan data show, in 2020, 1659 new cases of oropharyngeal cancer were diagnosed and registered in Poland [19].

The role of HPV in the development of OPSCC has been well-documented [2,3]. Very interesting results regarding the role of EBV in OPSCC were presented by Carpen et al. [3]. These researchers studying EBER (EBV-encoded small RNA) expression in HPV-negative OPSCC observed correlations between EBER expression and worse prognosis, which the authors believe is an interesting new observation regarding the involvement of EBV in OPSCC. The above-mentioned authors suggest that EBER expression as a marker of latent EBV infection may have prognostic significance.

Similar to other members of the *Herpesviridae* family, EBV has the ability to remain in a latent state, periodically reactivating to the lytic phase, which is important for cancer development [20]. Proteins of the lytic phase of the EB virus play a key role in oncogenesis, which has already been well-documented [21,22]. In the latency, the following proteins are expressed: EBNA1 (Epstein–Barr nuclear antigen 1), latent membrane protein 1 and 2 (LMP1, LMP2), Epstein–Barr virus-encoded RNA 1 and 2 (EBER1 and EBER2) and BamHI-A rightward transcripts (BART) [23].

EBNA1, the only protein synthesized in all EBV-dependent cancers, is responsible for viral replication [24]. It also has the ability to immortalize cells, cause DNA damage and change signaling pathways [10]. Moreover, EBNA1 can increase the expression of LMP1.

LMP1, synthesized by the BLNF1 gene, affects various processes occurring in the cell, changing their course. By modulating the tumor microenvironment (TME), it influences tumor transformation, proliferation, angiogenesis and metastasis [9,20]. Therefore, it is considered a prognostic indicator in NPC [25,26].

In the laboratory diagnosis of infections caused by EBV, serological tests (ELISA) are commonly used, which enable the identification of individual stages of this infection (primary, reactivation or past) [27,28]. Many authors have shown higher concentrations of EBV antibodies in the serum of NPC patients [29,30,31]. Anti-EBVCA antibodies, especially Ig A, have been considered as one of the important markers in screening for NPC [32,33].

Recently, many researchers have been focusing their attention on identifying diagnostic and prognostic markers in various cancers, including HNCs, while looking for the relationships between them and clinicopathological features.

Transcription factors (TFs) belong to a large group of proteins that, by stimulating or inhibiting gene transcription, are involved in a number of different human diseases, including carcinomas. One of the most important members of this group is nuclear factor kappa B (NF-κB), discovered in 1986 by Sen and Baltimore in the nuclei of mouse B lymphocytes as a constitutive protein essential in the transcription of the kappa immunoglobulin light chain [34,35].

The mammalian NF-κB group consists of five structurally similar members, including NF-κB1 (called p50), NF-κB2 (called p52), RelA (called p65), RelB and c-Rel [36,37]. NF-κB subunits have a structurally conserved region—the Rel homology domain (RHD), containing 300 amino acids in the N-terminal part of the polypeptide chain, which provides NF-κB with the ability to dimerize and bind DNA. NF-κB occurs in the form of a homo- or heterodimer. Each dimer binds specifically to a given DNA sequence.

The first, most widespread dimer identified was the p50/p65 [38]. Subsequently, many combinations of dimers existing as homodimers (p65/p65, c-Rel/c-Rel and p50/p50) or heterodimers (p52/c-Rel, p50/c-Rel, RelB/p50, RelB/p52, p65/c-Rel and p65/p52) have been described [36].

During the rest phase, NF-κB dimers occur in the cytoplasm in an inactive form by binding to inhibitors of κB (IκB). Various factors can activate NF-κB by degrading IkB, which leads to the translocation of dimers to the nucleus, where they attach to target genes.

NF-κB activation can occur in two ways, i.e., through the canonical (classical) pathway initiated by NF-κB1 (p50/p105) and the non-canonical (alternative) pathway initiated by NF-κB2 (p52/p100) [37,38,39].

The canonical NF-κB signaling pathway is activated by various stimuli, such as the tumor necrosis factor receptor (TNFR), various cytokine receptors, pattern recognition receptors (PRRs such as Toll-like receptors (TLRs)), B-cell receptors (BCRs) and T-cell receptors (TCRs). This pathway plays an essential role in inflammation and cell survival, as well as proliferation, transformation, angiogenesis and metastasis. The non-canonical NF-κB pathway, which is highly evolutionarily conserved, is controlled by genes that regulate homeostatic processes in the body, such as organogenesis of the lymphatic system, B cell survival and skeletal metabolism. This pathway can be activated by the following factors: tumor necrosis factor receptor agonists, including lymphotoxin β receptor (LTβR), B-cell activating factor (BAFF) and T-cell-dependent immune response factor (CD40).

**Figure 1:**
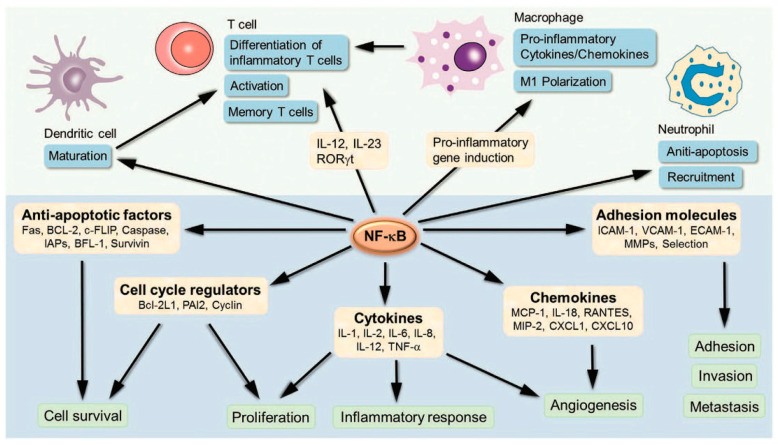
The relationship between NF-κB and other molecules [42].

NF-κB regulates the expression of various genes involved in the inflammatory process and in cell survival, proliferation and differentiation, as well as the immune response (both innate and adaptive) [37,39,40,41]. It is also an important factor in the activation of lymphocytes. Over 500 genes controlled by NF-κB have been described. This is well-illustrated by Figure 1, taken from the publication by Liu et al. [42].

Uncontrolled activation of the NF-κB signaling pathway is associated with the pathogenesis of various diseases, such as atherosclerosis, AIDS, diabetes, inflammatory bowel disease, stroke, muscle wasting, septic shock, rheumatoid arthritis and many autoimmune diseases [43]. Increased NF-κB activity has been described in many cancers [44].

The available global scientific literature provides abundant, well-documented evidence for the involvement of EBV in the development of NPCs. EBV is a cancer virus that stimulates oncogenesis by modifying many cell signaling pathways, especially NF-κB [45]. Viral products serve various strategies to activate the NF-κB cascade. It is well-documented that LMP1, the main oncoprotein of EBV, initiates activation of the NF-κB cascade. Overexpression of NF-κB was found in all NPC cases [45,46,47]. All publications discussed the role of NFkB in NPC. However, the subject of our research was OPSCC linked to EBV. Unfortunately, no such studies were found in the available scientific literature.

Therefore, in the present study, we endeavored to assess if NF-κB might function as a diagnostic and/or prognostic indicator in EBV-positive OPSCC. Pursuing this goal, we assessed the concentration of NF-κB in the serum of patients with EBV-positive OPSCC and EBV-negative OPSCC and compared them with the control group. Next, we investigated the relationship between the concentrations of NF-κB, grading and TN classification. Additionally, attempts were made to assess the possible correlation between NF-κB concentration and EBV antibody level, viral load and MMPs—MMP3 and MMP9. The precision of NF-κB has also been evaluated.

## 2. Materials and Methods

The current study is a continuation of our team’s previous research, which has already been published [48,49]. Hence, the groups studied and the methods used are the same. Therefore, a brief description of the examined patients and the methods used is presented below.

### 2.1. Description of the Study Groups

The study included one hundred and ten patients diagnosed and histopathologically confirmed with squamous cell carcinoma of the oropharynx (OPSCC), hospitalized at the Department of Head and Neck Otolaryngology of the University of Casemiro Pułaski in Radom. In this group, there were 58 EBV-positive and 52 EBV-negative patients. Two age groups were distinguished, i.e., 50–59 (average age 54.7; SD = 2.6) and 60–79 (average age 68.5; SD = 5.5). The baseline characteristics of all studied groups are presented in Table 1.

#### Principles of Selecting Patients for the Study Group

Two basic principles were followed, i.e., inclusive criterion—the presence of EBV DNA in the tumor tissue; exclusion criterion—the presence of HPV DNA in the tumor tissue.

Patient selection was consistent with the 8th edition of the American Joint Committee on Cancer TNM, which recommends screening all patients with OPSCC for HPV [50,51,52].

Histological evaluation was performed by experienced pathologists based on the guidelines of the World Health Organization, dividing tumors into the following types: well-differentiated (G1), moderately differentiated (G2) and poorly differentiated (G3) [53].

None of the patients had previously undergone either radio- or chemotherapy. No metastases were found in any of the patients.

The control group, selected according to demographic and epidemiological characteristics, consisted of 40 individuals from outpatient clinics in whom tumors had been ruled out.

### 2.2. Description of the Methods Used

Methods for isolation and subsequent detection of EBV DNA and HPV DNA in freshly frozen tumor tissue were performed as previously described [48].

NF-κB was detected in the serum of all studied subjects. Antibody, viral load and MMP3 and MMP9 results were taken from previous studies [48,49].

Serum NF-κB level was determined by the commercially available ELISA Assay Kit for Nuclear Factor Kappa B (NF-κB) SEB824HU (Cloud-Clone Corp. CCC, Houston, TX, USA) in accordance with the manufacturer’s instructions. The detection range is 0.156–10 ng/mL. The minimum concentration of NF-κB detected by this test is 0.059 ng/mL.

### 2.3. Statistical Analysis

The obtained results were subjected to statistical analysis using the following software: Tibco Statistica 13.3 (StatSoft, Kraków, Poland) and GraphPad Prism version 10.1.1. (San Diego, CA, USA). The normality of the distribution of continuous variables was checked using the Shapiro–Wilk test. Pearson’s chi-square test was used to assess the relationship between clinical and demographic parameters.

Differences between individual study groups were assessed using the Mann–Whitney U test and/or the Kruskal–Wallis test. However, any correlation between all studied variables was assessed using multiple linear regression analysis. In turn, the diagnostic accuracy of serum NF-κB concentration was checked by receiver operating characteristic (ROC) curve analysis.

### 2.4. Ethics

The study was conducted according to the guidelines of the Declaration of Helsinki and approved by the Medical University of Lublin Ethics Committee (No. KE-0254/295/2019, 26 September 2019). Written informed consent has been obtained from the patients to publish this paper.

## 3. Results

### 3.1. Assessment of the Incidence of NF-κB in Patients with Oropharyngeal Cancer Compared to the Control Group

At the beginning of our research, we analyzed the prevalence of NF-κB in the serum of the tested individuals. As shown in the data in Table 2, NF-κB was most often detected in patients with EBV-positive OPSCC (87.9%). This was a statistically significant difference both in comparison to the EBV-negative OPSCC patients and controls (*p* < 0.0001).

### 3.2. Assessment of Serum Level of NF-κB in Patients with Oropharyngeal Cancer Compared to the Control Group

**Figure 2:**
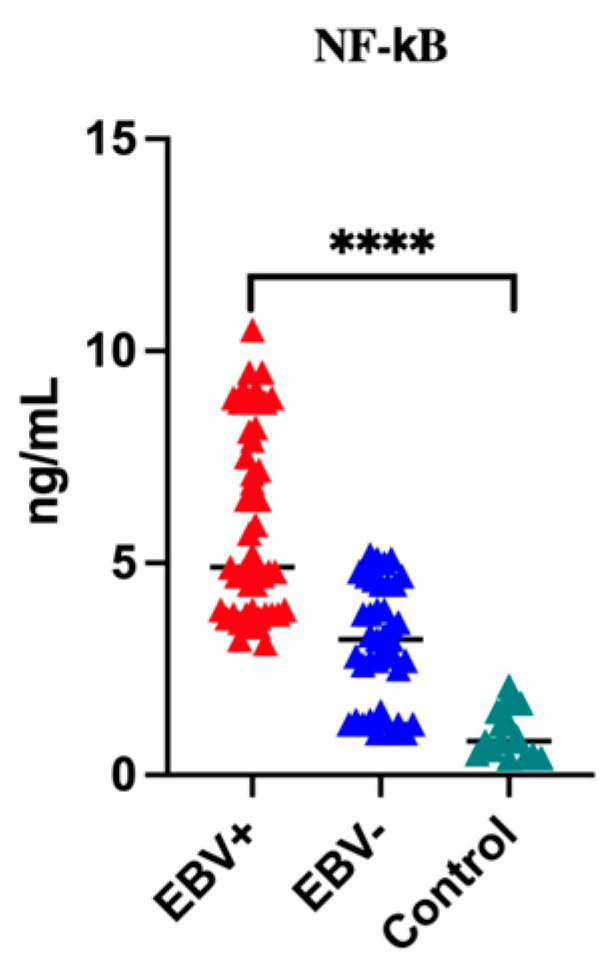
Serum level of NF-κB in all studied groups; Kruskal–Wallis Test; **** *p* < 0.0001.

Then, we compared the level of NF-κB in the serum of all patients, and we noticed that the level of the tested parameter differed between individual groups (Figure 2). The highest level of NF-κB was found in the serum of patients with EBV-related OPSCC (*p* < 0.0001). Detailed data for each study group are presented in Table 3.

### 3.3. The Frequencies of NF-κB in OPSCC Patients by Grading (G)

Only patients with EBV-related oropharynx carcinoma were qualified for further analyses. In this stage, the prevalence of NF-κB depending on the degree of tumor differentiation and TN classification was assessed (Table 4). The highest frequencies of NF-κB (statistically significant) were observed both in moderately (G2) and poorly (G3) differentiated carcinoma (*p* < 0.0001). In both groups, NF-κB was detected in all patients, while in G1 cases, it was detected in 63.2%. As the analysis showed, the incidence of NF-κB was related to the degree of tumor differentiation.

### 3.4. The Frequencies of NF-κB by TNM Classification

Then, we assessed the prevalence of NF-κB in relation to TNM classification. The small number of patients in the subgroups resulted in their combination. T and N features were analyzed in the following subgroups: T1–T2; T3–T4; N0–N1; N2–N3. The incidence of NF-κB was significantly dependent on the T trait and was higher in the group T3–T4. However, there was no difference in the incidence depending on the involvement of lymph nodes (N). These results are presented in Table 5.

### 3.5. Serum Level of NF-κB by Grading (G1–G3) and TN Classification

**Figure 3:**
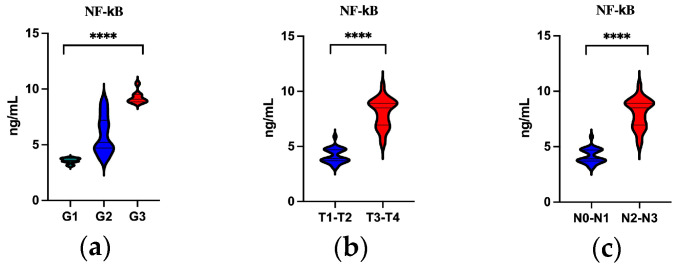
Serum level of NF-κB in EBV-positive OPSCC: (**a**) concentration of NF-κB by grading; (**b**) concentration of NF-κB by T stage; (**c**) concentration of NF-κB by N stage; Kruskal–Wallis test and Mann–Whitney test; **** *p* < 0.0001.

The analysis shows that the concentration of NF-κB in the serum of OPSCC patients depended both on the degree of tumor differentiation (grading) (Figure 3a) and on the T (Figure 3b) and N (Figure 3c) features. The highest concentration of this parameter was observed in poorly differentiated tumors as well as in more advanced clinical stages (T3–T4; N2–N3 groups).

### 3.6. Analysis of the Serum Concentration of NF-κB Depending on the EBV Load

**Figure 4:**
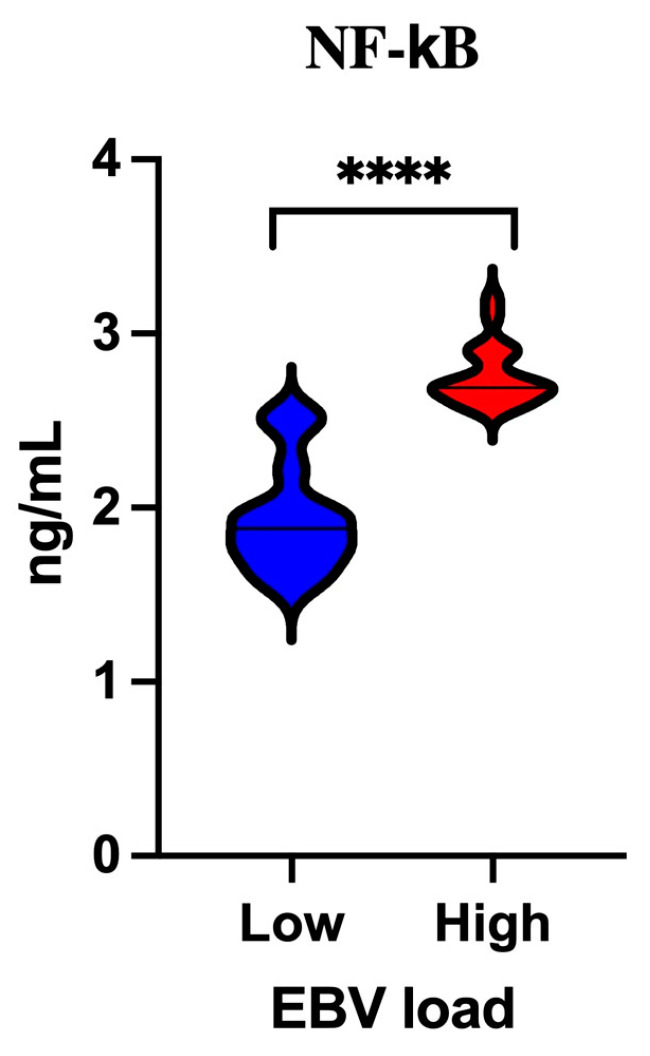
Serum concentration of NF-κB in relation to viral load; Mann–Whitney U Test; **** *p* < 0.0001.

In this step of our study, we analyzed the level of NF-κB in relation to viral load (Figure 4). EBV DNA has been detected in the tumor tissue of OPSCC patients; therefore, the viral load was semi-quantitatively determined based on the value of the cycle threshold (Ct) of the viral gene. Viral load was assumed to be high if the Ct value was above 38 and low if the Ct value was ≤38. Higher levels of NF-κB were detected in cases with high viral load (*p* < 0.0001).

### 3.7. Correlation between the Serum Level of NF-κB, EBV Antibodies, MMP3, MMP9 and Viral Load

**Figure 5:**
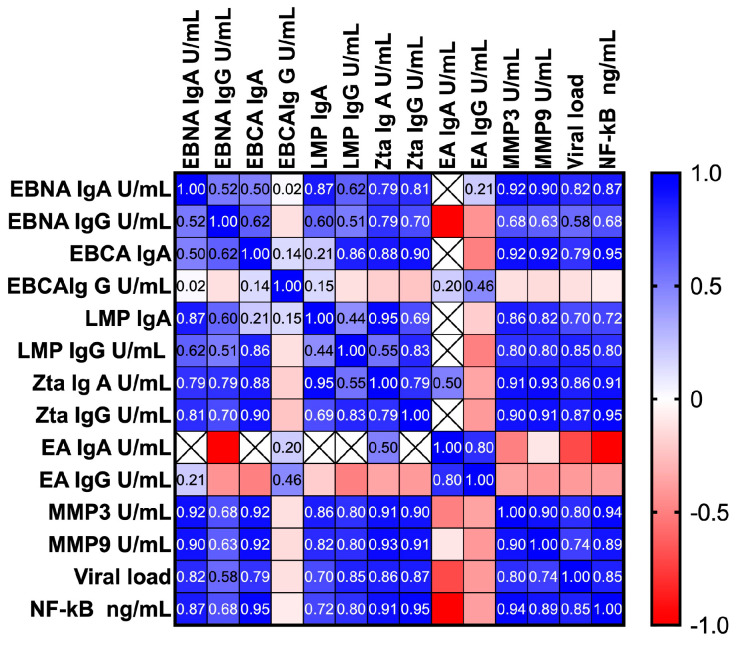
Correlation between the level of NF-κB, EBV antibodies, MMP3, MMP9 and viral load in EBV-related OPSCC patients. Spearman rank coefficients are presented as color intensities. The closer Rs is to +1 or −1, the stronger the correlation. A perfect positive correlation is +1 (blue), and a perfect negative correlation is −1 (red).

We also considered the possibility of correlation with variables analyzed in previous studies, such as EBV antibodies, MMP3, MMP9 and viral load. The analysis carried out using the multiple linear regression method showed a high correlation between the studied variables (Figure 5). However, no relationship was detected between the levels of NF-κB and EA antibodies in either the IgA or IgG classes. Detailed analysis results are presented in Table 6.

### 3.8. Assessment of the Diagnostic Accuracy of Serum NF-κB Measurements Using ROC Analysis (Receiver Operating Characteristic)

**Figure 6:**
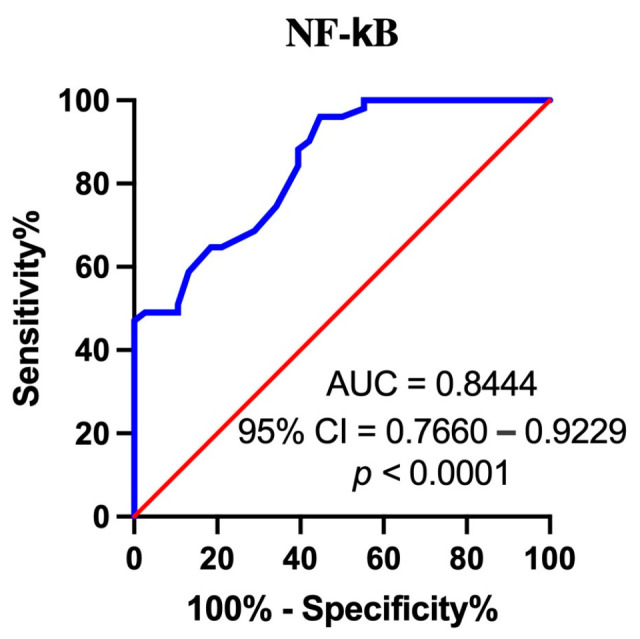
ROC analysis for NF-κB. Area under curve (AUC) analysis showed that NF-κB is the most sensitive and specific biomarker for identifying EBV-infected OPSCC subjects. NF-κB concentration (ng/mL) is marked with a blue line.

The last point of our studies was the assessment of the diagnostic accuracy of NF-*κ*B. We wanted to investigate whether serum NF-κB could serve as a diagnostic and/or prognostic indicator in EBV-related OPSCC. ROC analysis was used for this purpose. By analyzing AUC (area under the curve), NF-κB concentration was compared in the group of patients with EBV-related OPSCC with the group of EBV-negative individuals (Figure 6). The analysis performed showed that the NF-κB level was a sensitive and specific parameter to identify patients with EBV-related OPSCC (AUC = 0.8444; Std. error 0.0434; 95% CI 0.7660–0.9229; *p* = 0.0001). The obtained results confirm the diagnostic accuracy of NF-κB; therefore, its identification in serum seems to be a promising biomarker for the detection of EBV-related oropharyngeal carcinoma.

## 4. Discussion

The involvement of the EBV virus in the pathogenesis of NPC has already been well-documented in the scientific literature [45]. Although EBV causes asymptomatic, lifelong infection in over 90% of the world’s population, only a small percentage develop NPC [54]. There are large disproportions in the incidence and mortality depending on the geographical region. In endemic regions, EBV is associated with 95% of NPCs and 100% of deaths, while in non-endemic areas, it is associated with 20% and 80%, respectively. In the Polish population, the dominant type of HNC is OPSCC.

According to global data from 2020, EBV is estimated to be associated with 239,700–357,900 new cancer cases and 137,900–208,700 cancer deaths [55]. As reported in the article by de Martel et al. [56], 84.6% of all newly diagnosed cases of NPC are associated with EBV.

Due to such a high involvement of EBV in the development of HNCs, precise diagnostic methods are necessary, and also finding targets for new anticancer drugs. Early diagnosis and effective therapy are the fundamental challenge for modern oncology. Hence, many researchers focus on the search for new or improved biomarkers. Biomarkers, although a relatively new clinical tool in the diagnosis and prognosis of various cancers, can significantly improve the therapeutic process. Therefore, it seems that simultaneous detection of several biomarkers may be diagnostically useful. Due to differences in specificity and sensitivity, one diagnostic strategy is to test for several biomarkers [57].

In the case of NPC, the combined testing of several serum EBV antibodies and plasma EBV DNA by PCR is recommended to assess prognosis and develop treatment strategies [54]. Our research fits into this global trend.

In one of the first studies by our research team on the frequency of various viruses with oncogenic potential in oropharyngeal cancer, we detected EBV in 53.3% of patients. Since then, we have been analyzing various aspects of this, also examining the presence of selected biomarkers in serum, saliva and cancer tissue. Therefore, the concept of a series of studies on the diagnostic and prognostic usefulness of these biomarkers was born.

Hence, in our first study, we focused on assessing the incidence and titer of anti-Zta and anti-LMP1 antibodies, concluding that combined tests increase diagnostic precision [48]. In turn, in the second study, we analyzed the titers of MMP3 and MMP9, demonstrating their utility as biomarkers in EBV-related OPSCC [49]. The literature review shows that the presented studies are the first to examine the above-mentioned biomarkers among Polish patients with EBV-related OPSCC. There have been no such studies so far.

Many different factors are involved in the etiopathogenesis of HNC in general but also in OPSCC. As many studies indicate, the microbiome plays a significant role in oncogenesis [58,59]. The influence of environmental and lifestyle factors (smoking and/or alcohol consumption) has already been well-documented [15,16,17]. However, persistent infections with oncogenic viruses can also significantly influence oropharyngeal cancer initiation and progression.

Furthermore, various genetic factors may contribute to the development of EBV-related cancer, only some of which have been identified so far [60]. Increasing scientific evidence indicates that EBV, by affecting the TME, creates an immunosuppressive environment conducive to the development of EBV-related malignancies. In the TME, a constantly modified, complex ecosystem, various molecules interact to promote tumor growth and progression [24]. On the other hand, the interaction of body cells and viral factors creates an environment favorable to oncogenesis [17].

Viruses, bacteria and other pathogens that make up the human body’s microflora participate in various physiological and pathological processes. However, changes in the composition of microflora may promote the development of tumors [59]. Tumoral microflora can influence cancer cell physiology, as well as the immune response, through various signaling pathways, including NF-κB.

Viral DNA stimulates the expression of pro-inflammatory cytokines and interferons, which contribute to the development of various diseases by modulating the immune system [60].

As already mentioned in the Introduction, nuclear factor kappa B (NF-κB) plays a key role in inflammation and the immune response regulating various genes [42,43]. Dysregulation of this protein may also be caused by latent EBV infection. NF-κB overexpression inhibits apoptosis of cancer cells, leading to cancer proliferation and progression. In the development of NPC, NF-κB becomes impaired, as indicated by NF-κB overexpression in almost all cases of this cancer [46,47].

EBV has the ability to integrate its genome into the genome of the infected cell, establishing a latent phase, and may be periodically reactivated under the influence of various stressors [61]. To prevent cancers associated with EBV reactivation, a detailed understanding of these mechanisms is necessary.

Even though our research concerns a different tumor location, the results obtained are similar. In our study, NF-κB was most frequently detected in the serum of EBV-positive OPSCC patients. Moreover, we showed significantly higher levels of NF-κB in the serum of this group of patients. When assessing the prevalence of NF-κB depending on tumor grading and TN classification, we observed the highest frequency of NF-κB in both moderately (G2) and poorly differentiated tumors (G3). Additionally, the incidence of NF-κB was higher in more advanced stages (T3–T4). However, there was no difference in the incidence depending on the involvement of lymph nodes (N).

Analyzing the level of NF-κB depending on the stage of clinical advancement, a significant relationship was observed, i.e., the concentration of this protein in the serum depended on the degree of tumor differentiation and T and N features. In addition, significantly higher NF-κB concentrations were observed in the serum of patients with high viral load.

While reviewing the world literature on this topic, we came across very interesting research by Zhou et al. [62], which is similar to ours in its assumption. Namely, these authors analyzed the clinicopathological significance of NF-κB p65 and IKKβ protein and mRNA expression in NPC patients in China. The results they obtained showed that overexpression of NF-κB in NPC tissues resulted in a worse prognosis. Therefore, they suggested that NF-κB may be a therapeutic target for NPC. These authors observed significantly higher NF-κB p65 expression in advanced T stage (T3–T4) and lymph node metastasis. However, they did not find any relationship between NF-κB p65 expression and the degree of tumor differentiation.

Serological assessment of anti-EBV antibody titer, as shown by numerous studies, is commonly used in the early diagnosis of NPC [63,64].

As mentioned above, there are two phases in the EBV life cycle, i.e., the latent phase and the lytic phase. The activator of the lytic cycle is the Zta protein synthesized by EBV [45]. There is a mutual negative interaction between lytic trans-activator Zta and NF-κB. Some studies indicate that NF-κB (p65/RelA) promotes latency by suppressing Zta [65]. In turn, Zta can inhibit NF-κB (p65 subunit) and thus initiate the lytic phase [66].

LMP1, the main oncoprotein of EBV, is found in 80–90% of NPC cases [67]. As studies conducted in NPC-positive cell lines have shown, NF-κB inhibitors lead to EBV reactivation and subsequent cell death. LMP1 is an activator of NF-κB signaling in NPC, and as reserchers have shown, high expression of this oncoprotein is observed in 25.7% of cases [68]. It, therefore, appears that dysregulation of this pathway through genetic mutations or viral infection is crucial in the development of NPC.

Moreover, many changes in the TME occur with the participation of MMPs. Therefore, their reduced or excessive expression may result in cancer initiation and progression [69]. MMP3 and MMP9 play a significant role in this process, and their high levels occur in advanced clinical stages, as numerous studies have shown [70,71,72,73,74]. Many MMPs have been shown to be overexpressed in head and neck cancer: MMP1, MMP2, MMP3, MMP7, MMP8, MMP9, MMP10, MMP11, MMP13 and MMP14 [75]. We chose these two MMPs for our research. There would not be enough clinical material to test a panel of metalloproteinases. However, the main reason for this choice was the inspiration from the research results of other authors indicating the role of these MMPs in the development of NPC. We wanted to test whether these selected MMPs might play a similar role in EBV-related oropharyngeal cancer.

Due to the interconnection of antibodies, MMPs and the NF-κB, we tried to evaluate the relationship between these parameters. To achieve this goal, we utilized findings from our aforementioned prior studies to examine potential correlations among NF-κB levels, anti-EBV antibodies, MMP3, MMP9 and viral load. Following statistical analysis employing multiple linear regression, we confirmed a strong correlation among them. Nonetheless, no correlation emerged between NF-κB levels and EA antibodies.

In the concluding phase of our study, we wanted to check the precision of NF-κB, i.e., whether NF-κB in serum can be a good diagnostic and/or prognostic indicator in the early diagnosis of OPSCC. The results obtained with the ROC method confirmed its accuracy, suggesting that this protein may be used as a biomarker in screening patients with OPSCC.

Many researchers pay attention to the crucial role of the NF-κB signaling pathway in the development of NPC [76,77,78]. EBV, and more precisely, its oncoproteins, initiate tumor formation by modulating many signaling pathways, such as NF-κB, JAK/STAT, Wnt/β-catenin, PI3k/Akt/mTOR and EGFR/MAPK. The complex molecular mechanisms involved in the pathogenesis of NPC, with particular emphasis on signaling pathways, have been thoroughly described by Richardo et al. [79].

## 5. Limitations of Own Research

We are aware that our current study, like the previous one, has certain shortcomings. The study group was not very large, which is due to the low incidence of this type of cancer in our region. This, in turn, was the reason for the small numbers in individual subgroups. Therefore, for the purpose of statistical calculations, they were combined and analyzed as: T1–T2, T3–T4, N0–N1 and N2–N3 features. EBV DNA was detected in tumor tissue and not in plasma, as suggested by many authors. This procedure resulted from the fact that we were measuring many different parameters in the same samples, and neither serum nor plasma was available anymore.

These limitations will be supplemented in the future with specially designed research on a larger number of patients, which will allow for the verification of the observed trends. Nevertheless, it seems that despite these limitations, the presented research is fully justified. In addition, the results obtained encourage us to conduct further research. In our current study, the assessment of serum NF-κB levels was performed after diagnosis. Perhaps in the future, it would be worth determining this level in other periods of the natural course of the disease. It would then be possible to assess the possible dynamics of this biomarker concentration both before, during and after therapy.

The NF-κB pathway has various functions in the human body; therefore, inhibition of this pathway may cause side effects in patients. Unfortunately, finding an inhibitor of the NF-κB pathway is difficult [80]. Therefore, most likely, despite numerous studies, none of the NF-κB inhibitors has been approved for clinical practice yet. Nevertheless, further studies are necessary to understand the exact signaling pathways and the interactions of various molecules involved in them, which may constitute new targets in the development of therapy for NPC and perhaps also OPSCC.

## 6. Conclusions

Since the discovery of NF-κB, there has been significant progress in understanding numerous and complex signaling networks. In particular, inhibition of NF-κB appears to be a future therapeutic strategy, especially for cancer. Although many issues remain unexplained, the results obtained so far constitute the basis for continuing this line of research.

In conclusion, we have established that the serum NF-κB levels were significantly higher in the EBV-positive OPSCC patient group. Its concentration was positively correlated with the progression of OPSCC and additionally with the EBV antibody titer, viral load and MMP3 and MMP9 levels. Diagnostic precision was confirmed using ROC analysis.

We hope that our study will expand knowledge about the role of NF-κB in the pathogenesis of OPSCC and, consequently, the usefulness of this protein as a biomarker in both diagnosis and prognosis while providing a rational basis for future research in this area.

## Figures and Tables

**Table 1:** Characteristics of the study and control groups.

		EBV				*p*	Total Patients		Control Group		*p*
		Positive		Negative							
		N	%	N	%		N = 110	%	N = 40	%	
Sex	Female	8	13.8	7	13.5	0.9999	15	13.8	6	15.0	0.7957
	Male	50	86.2	45	86.5		95	86.2	34	85.0	
Age	50–59	27	46.6	24	46.2	0.1116	59	53.4	21	52.5	0.9999
	60–79	31	53.4	28	53.8		51	46.6	19	47.5	
Place of residence	Urban	41	70.7	36	69.2	0.1667	77	70.7	28	70.0	0.9999
	Rural	17	29.3	16	30.8		33	29.3	12	30.0	
Smoking	≤10 >10	28 10	48.3 17.2	25 10	48.1 19.2	0.8427	53 20	48.3 18.2	16 10	40.0 25.0	0.9999
	No	20	34.5	17	32.7		37	34.5	14	35.0	
Alcohol abuse	≤10 >10	18 10	31.1 17.2	15 10	28.8 19.3	0.9834	53	48.3	19	47.5	0.9999
	No	30	51.7	27	51.9		57	51.7	21	52.5	
G	G1	19	32.8	17	32.7	0.9997					
	G2	30	51.7	27	51.9						
	G3	9	15.5	8	15.4						
T	T1	7	12.1	8	15.4	0.9505					
	T2	27	46.6	22	42.3						
	T3	16	27.6	15	28.8						
	T4	8	13.7	7	12.1						
	N0	23	39.7	22	42.3						
N	N1	11	19.0	10	19.2	0.9844					
	N2	14	24.1	11	21.2						
	N3	10	17.2	9	17.3						
M	M0	58	100.0	52	100.0						

**Table 2:** Prevalence of NF-κB in the studied groups (%).

	EBV+ N = 58 N (%)	*p*-Value	EBV− N = 52 N (%)	Control Group N = 40 N (%)	*p*-Value
NF-κB	51 (87.9)	<0.0001 *	28 (53.8)	15 (37.5)	<0.0001 *

* statistically significant; Pearson’s chi-square test; Fischer’s exact test.

**Table 3:** The serum level of NF-κB in studied groups (ng/mL).

Group	Mean	Minimum	Maximum	SD	*p*-Value
EBV+ EBV− Control	5.9 3.1 1.01	3.1 1.0 0.4	10.5 5.2 2.1	2.2 1.4 0.6	<0.0001 *

* statistically significant; Kruskal–Wallis test.

**Table 4:** The prevalence of NF-κB in EBV-positive OPSCC patients by grading (%).

Parameter (%)	G1 N = 19 N (%)	G2 N = 30 N (%)	G3 N = 9 N (%)	*p*-Value
NF-κB	12 (63.2)	30 (100.0)	9 (100.0)	0.0003 *

* statistically significant; Pearson’s chi-square test; Fischer’s exact test.

**Table 5:** The prevalence of NF-κB in OPSCC patients by TNM Classification (%).

**Parameter**	**T1–T2** **N = 34** **N (%)**	**T3–T4** **N = 24** **N (%)**	***p*-Value**
NF-κB	27 (79.4)	24 (100.0)	0.0343 *
**Parameter**	**N0–N1** **N = 34** **N (%)**	**N2–N3** **N = 24** **N (%)**	***p*-Value**
NF-κB	31 (91.2)	24 (100.0)	0.2595

* statistically significant; Pearson’s chi-square test; Fischer’s exact test.

**Table 6:** Correlation between the level of NF-κB, EBV antibodies, MMP3, MMP9 and viral load in EBV-positive OPSCC patients.

Parameter	95% CI of rs	*p*-Value
EBNA IgA U/mL	0.6974–0.9509	<0.0001 *
EBNA IgG U/mL	0.4711–0.8150	<0.0001 *
EBVCA IgA	0.8671–0.9813	0.0001 *
EBVCA IgG U/mL	−0.3771–0.2295	0.5997
LMP1 IgA U/mL	0.3656–0.8907	0.0007 *
LMP1 IgG U/mL	0.6133–0.8983	<0.0001 *
Zta IgA U/mL	0.8153–0.9603	<0.0001 *
Zta IgG U/mL	0.8957–0.9783	<0.0001 *
EA IgA U/mL	0.7982–0.1806	0.2333
EA IgG U/mL	−0.7318–0.1449	0.1388
MMP3 U/mL	0.9018–0.9683	<0.0001 *
MMP9 U/mL	0.8128–0.9376	<0.0001 *
Viral load	0.7482–0.9140	<0.0001 *

* statistically significant.

## Data Availability

Due to privacy and ethical concerns, the data used in this study are available from the corresponding author upon reasonable request.
